# Are two beneficial mutations (p.Q249R and 90-bp Indel) within the ovine *BMPRIB* gene associated with growth traits?

**DOI:** 10.3389/fvets.2023.1280548

**Published:** 2024-04-05

**Authors:** Hongwei Xu, Nazar Akhmet, Yunyun Luo, Zhenggang Guo, Chuanying Pan, Enliang Song, Nurlan Malmakov, Zhanerke Akhatayeva, Xianyong Lan

**Affiliations:** ^1^College of Life Science and Engineering, Northwest Minzu University, Lanzhou, Gansu, China; ^2^Key Laboratory of Animal Genetics, Breeding and Reproduction of Shaanxi Province, College of Animal Science and Technology, Northwest A&F University, Yangling, Shaanxi, China; ^3^Bijie Animal Husbandry and Veterinary Science Research Institute, Bijie, Guizhou, China; ^4^Shandong Key Lab of Animal Disease Control and Breeding, Institute of Animal Science and Veterinary Medicine, Shandong Academy of Agricultural Sciences, Jinan, China; ^5^Scientific Research Institute of Sheep Breeding Branch, Kazakh Scientific Research Institute of Animal Husbandry and Fodder Production, Mynbaev, Almaty Region, Kazakhstan

**Keywords:** sheep, FecB (*BMPRIB*), polymorphism, growth traits, MAS

## Abstract

**Background:**

The problem of achieving economic efficiency in sheep breeding can be largely solved by increasing sheep productivity. Recently, the *BMPRIB* gene has been revealed by GWAS as a potential candidate gene for sheep body morphometric traits. Therefore, the present study aimed to investigate whether genetic polymorphisms (p.Q249R SNP and 90-bp deletion) in the *BMPRIB* gene are associated with sheep growth traits.

**Methods:**

PCR-based genotyping was performed on 1,875 sheep, including 1,191 Guiqian semi-fine wool (GQSFW), 560 Luxi Blackhead (LXBH), 55 Lanzhou fat-tailed (LZFT), and 69 Weining (WN) sheep. Genotype–phenotype association was assessed using the independent samples *t*-test and ANOVA. The significance level was set at α_original_ < 0.05. The threshold *p*-value for significance was adjusted after correction for multiple comparisons using the Bonferroni correction.

**Results:**

After the Bonferroni correction, it was found that individuals with FecB^+^/FecB^+^ genotypes of the p.Q249R had significantly better growth traits in LXBH ewe lambs, including the body length, chest width, paunch girth, cannon circumference, and hip width (*P*<0.0005). Meanwhile, associations were observed between 90-bp deletion polymorphism and several growth traits (body length, body height, chest depth, and canon circumference) in GQSFW ewe adults after the Bonferroni correction (*P* < 0.0002), and individuals with the “DD” genotypes had greater growth traits.

**Conclusion:**

Our findings align with the experimental observations from GWAS, which identified the *BMPRIB* gene as a potential candidate gene for body measurement traits. These findings not only confirm the previous study's results but also expand on them. Therefore, further investigations regarding the impact of *BMPRIB* polymorphisms on growth traits are necessary in other sheep breeds.

## 1. Introduction

The knowledge of growth patterns and their use in the management of individual development of animals is an additional reserve for improving their productivity and livestock production (1). The advancements in molecular genetics include creating opportunities for identifying key genes, the polymorphism of which makes an important contribution to the realization of economically useful traits in livestock (2). The presence of diverse allelic variants as well as polymorphisms of genes and genotypes is a prerequisite for **successful** breeding (3).

The *BMPRIB (Bone morphogenetic protein receptor type 1B)* gene is one of the key candidate genes that control the ovulation rate and a subsequent increase in fecundity, which were confirmed by a comprehensive series of experiments using different sheep breeds globally (4, 5). The major functions attributed to *BMPRIB* are cell proliferation, differentiation, and apoptosis (6). Of interest is that the highest expression of the *BMPRIB* gene was found in the hypothalamus of Small Tail-Han sheep (7). Moreover, the *BMPRIB* gene contains the candidate variation p.Q249R (also known as Fec^B^ or g.746A>G) that encodes a member of the type-I bone morphogenetic protein (BMP)—the receptor family of transmembrane serine/threonine kinases that has an important function in sheep reproduction. Moreover, several pieces of evidence indicate that the current mutation is associated with litter size in various sheep breeds (8–10) because the essence of the action of this locus is to increase the ovulation rate, which leads to an increase in sheep litter size (11–13). A more recent study showed that serum concentrations of follicle-stimulating hormone (FSH), luteinizing hormone (LH), estrogen (E2), and progesterone (P4) can vary significantly depending on the FecB genotype (14). The study results of numerous investigations showed that FecB^B^ is one of the significant variants that can be used as a molecular genetic marker for the early selection of high-yielding ewes (15, 16).

The same phenotypic trait is usually determined by many genes. Consequently, many quantitative trait loci (QTLs) are associated with a particular trait and are often located on different chromosomes. QTLs that explain the variation in phenotypic traits help to form the genetic structure of the phenotype (1, 3). Of interest is that GWAS revealed quantitative trait loci (QTLs) for body measurement traits and the *BMPRIB* gene as a potential candidate gene for sheep growth traits in Argali, Tibetan, and hybrid sheep (17). Moreover, Gootwine et al. (18) revealed that p.Q249R was associated with birth weight and mature weight of ewes. Furthermore, researchers have explored the effect of this variant on the growth of lambs in Indian Chhotanagpuri mutton sheep. A previous study revealed that growth traits of animals with homozygous BB genotypes were significantly higher than those with other genotypes (19). Furthermore, the body weights and growth performance of BB and B+ lambs were higher (*P* < 0.05) than those of ++ lambs in Chinese Merino sheep (20).

Recently, p.Q249R and 90-bp deletion were identified in Guiqian semi-fine wool (GQSFW), Luxi Blackhead (LXBH), Lanzhou fat-tailed (LZFT), and Weining (WN) sheep (21); however, no genetic association studies have been carried out between these polymorphisms and body morphometric traits in these sheep breeds. The GQSFW sheep is a type of wool and meat hybrid breed developed under the natural ecological conditions of high altitude, cold, and humid regions, and its breeding will improve the production level of semi-fine wool sheep and contribute to the development of the local rural economy in China (21). The action of FecB has been well-studied in the best-known high-prolific sheep breeds in China, such as the Hu and the Small-Tail Han breeds (5), but this major determinant of fecundity might affect other breeds as well. Thus, identification of the major fecundity *BMPRIB* gene in GQSFW, LXBH, LZFT, and WN breeds can help make considerable improvements in the breeding program and increase the production of mutton. In practical breeding, the involvement of the same single nucleotide polymorphic variants (SNPs) or insertion/deletions (InDels) in the formation of various phenotypic variability is highly valuable (22). Therefore, this study aimed to explore the relationship of 90-bp deletion and p.Q249R mutation with growth traits in various sheep populations.

## 2. Materials and methods

### 2.1. Animals and their phenotypic data

To explore the polymorphisms in the *BMPRIB* gene, a total of 1,875 sheep were randomly selected. In this study, the samples of the LXBH sheep (*n* = 560; Liaocheng, Shandong Province, China), GQSFW sheep (*n* = 1,191; Guizhou, China), LZFT sheep (*n* = 55; Yongjing, Gansu Province, China), and WN (*n* = 69; Weining, Guizhou, China) sheep were studied. Reports regarding general characteristics of sheep flocks, such as age, feeding, and management conditions, were provided in previous studies (1–3). Briefly, flocks were kept under identical feeding and management conditions. The age range of LXBH lambs, weaners, adult ewes, and rams was 1-18 months and older (1). For LZFT ewes and rams, the age range was 26 years old, while for GQSFW, it was 1-8 years old. Finally, the age range of WN ewes and rams was 2 months to 4 years.

The following growth traits such as body length (BL), body height (BH), body weight (BW), cannon circumference (CaC), chest depth (ChD), chest circumference (ChC), chest width (ChW), height at the hip cross (HHC), hip-width (HW), and paunch girth (PG) were recorded. Additionally, body length index (BLI), body trunk index (BTI), limb length index (LLI), chest width index (ChWI), chest circumference index (ChCI), and cannon circumference index (CaCI) were calculated (1, 3).

### 2.2. Genomic DNA extraction and genotyping

Genomic DNA (gDNA) of ear tissues or whole blood samples was extracted using a phenol-chloroform extraction method. The concentration and purity of extracted DNA were measured using a NanoDrop 2000 (Thermo Fisher Scientific, Waltham, MA, USA). Then, every sample was diluted to a standard concentration of 20 ng/μl and stored at -40°C.

Polymerase chain reaction (PCR)-based genotyping processes were described in previous studies (1, 23). For the assessment of polymorphisms, a touch-down (TD)-PCR method was utilized (1). In brief, the TD-PCR was carried out in a 13-μl full volume consisting of 0.5 μl of genomic DNA, 0.3 μl each of forward and reverse primers, 6.5 μl of 2×Eco Taq PCR Supermix, and 5.4 μl of ddH_2_O. The amplification conditions consisted of an initial denaturation step at 95°C for 5 min, followed by 18 cycles of denaturation at 94°C for 30 s, annealing at 68°C (with a reduction of 1°C per cycle) for 30 s, and extension at 72°C for 40 s, followed by another 30 cycles at 94°C for 30 s, at 50°C for 30 s, and at 72°C for 40 s. A final extension step at 72°C for 10 min was carried out. Ultimately, the PCR products were sent for sequencing (Sangon Biotech Co., Ltd. Xi'an, China).

### 2.3. Statistical analysis

Genetic parameters were computed according to previous studies (1, 3). We assessed genotype–phenotype association using the independent samples *t*-test (=2 group) and ANOVA in the SPSS software (Version 25.0, IBM Corporation, New York, USA). The significance level was set at α_original_ < 0.05. The threshold *p-*value for significance was adjusted after correction for multiple comparisons using the Bonferroni correction (α_altered_ = *P*/n, where *P* = 0.05, n = number of sheep). The formula that was used to determine the correlation between the growth traits of sheep and the varying genotypes of indels was reported in previous studies (1, 3).

## 3. Results

### 3.1. Genetic parameters analysis

The description of the analyzed 90-bp deletion and p.Q249R polymorphisms and population genetic parameters in LXBH (*n* = 560), LZFT (*n* = 55), and WN (*n* = 69) sheep are provided in the study by Zhang et al. (16).

Meanwhile, this study included a large sample size (*n* = 1,191) of the GQSFW sheep. For the Del-90-bp site, it had the three [insertion/insertion (II); insertion/deletion (ID); deletion/deletion (DD)] genotypes in GQSFW flocks. We also observed a deviation from Hardy–Weinberg Equilibrium (HWE). In addition, the mean polymorphism information content (PIC) of the Del-90-bp marker was 0.193, suggesting that genetic diversity is not abundant in GQSFW sheep. Detailed information on genetic parameters for the Del-90-bp indel is given in Table 1.

**Table 1:** Genetic diversity parameters for Del-90-bp in four sheep breeds.

**Breeds**	**Sample sizes**	**Genotypic frequencies**			**Allelic frequencies**		**HWE *P* values**	**Genetic parameters**				**Source**
		**II**	**ID**	**DD**	**I**	**D**		**Ho**	**He**	**Ne**	**PIC**	
GQSFW	1,191	0.777	0.198	0.024	0.877	0.123	*P* < 0.05	0.784	0.216	1.276	0.193	This study
LXBH	560	0.518	0.455	0.027	0.746	0.254	*P* < 0.05	0.621	0.379	1.611	0.307	(16)
WN	69	0.841	0.145	0.015	0.913	0.087	*P* > 0.05	0.841	0.159	1.189	0.146	
LZFT	55	0.236	0.455	0.309	0.464	0.536	*P* < 0.05	0.503	0.497	1.990	0.374	

II, insertion/insertion; ID, insertion/deletion; DD, deletion/deletion; HWE, Hardy-Weinberg equilibrium; Ho, homozygosity; He, heterozygosity; Ne, effective allele numbers; PIC, Polymorphism information content.

### 3.2. Association analysis of the p.Q249R mutation and sheep growth traits

The results of this study confirmed the previous finding of the associations between *BMPRIB* polymorphisms and growth traits in sheep. According to the results of independent samples *t*-tests, for p.Q249R locus, LXBH ewe lambs with FecB^+^/FecB^+^ genotype had significantly better growth traits, including body length, chest width, paunch girth, cannon circumference, and hip width in ewe lambs (*P* < 0.0005) (Table 2). However, other body measurement traits had no significant relation after the Bonferroni correction; therefore, they are not listed in the tables.

**Table 2:** Association of the p.Q249R mutation with growth traits in ewe lamb of LXBH.

**Gender**	**Age/stage**	**Growth traits**	**Observed genotypes**		***P*-values**
			++	**B**+	
Ewe	Lamb	BL (cm)	60.72 ± 0.6 (*n* = 83)	55.21 ± 0.7 (*n* = 24)	7.8777^*^10^−8^
		ChW (cm)	15.16 ± 0.3 (*n* = 83)	13.27 ± 0.4 (*n* = 24)	0.000365
		PG (cm)	88.71 ± 1.0 (*n* = 83)	79.25 ± 1.8 (*n* = 24)	0.000054
		CaC (cm)	7.75 ± 0.1 (*n* = 83)	7.08 ± 0.1 (*n* = 24)	0.000078
		HW (cm)	14.03 ± 0.3 (*n* = 80)	11.93 ± 0.3 (*n* = 23)	0.000012

BL, body length; ChW, chest width; CaC, cannon circumference; PG, paunch girth; HW, hip width; With the *p*-value corrected for multiple comparisons (α_altered_ = *P*/n, where *P* = 0.05, n = number of sheep) and after the Bonferroni correction, no association withstands (*P* < 0.0005). Genotypes were omitted because the number size was less than three or not detected.

### 3.3. Association analysis of the 90-bp deletion and sheep growth traits

**Figure 1:**
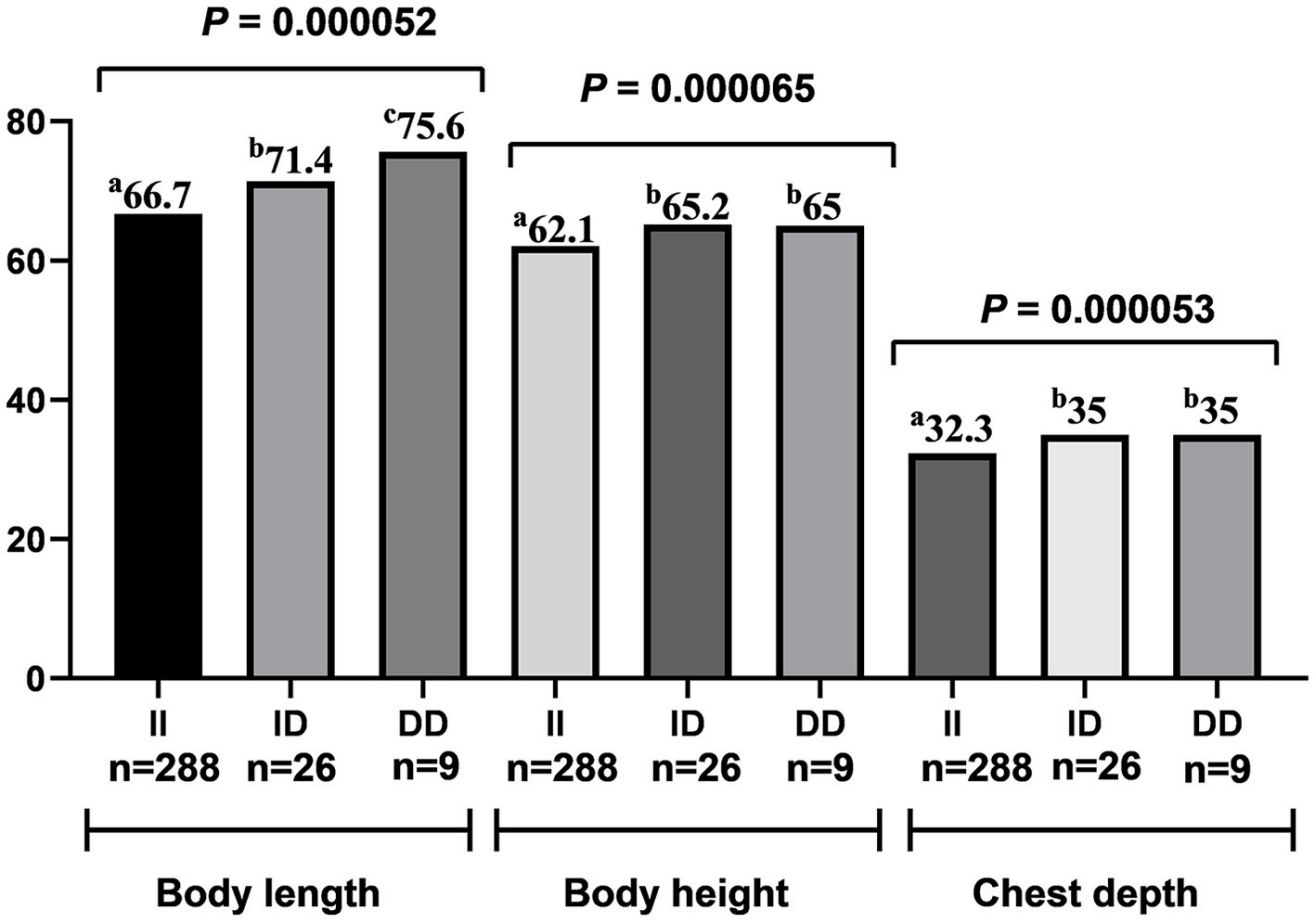
Association of the Del-90-bp with growth traits in adult ewes of the GQSFW sheep.

**Figure 2:**
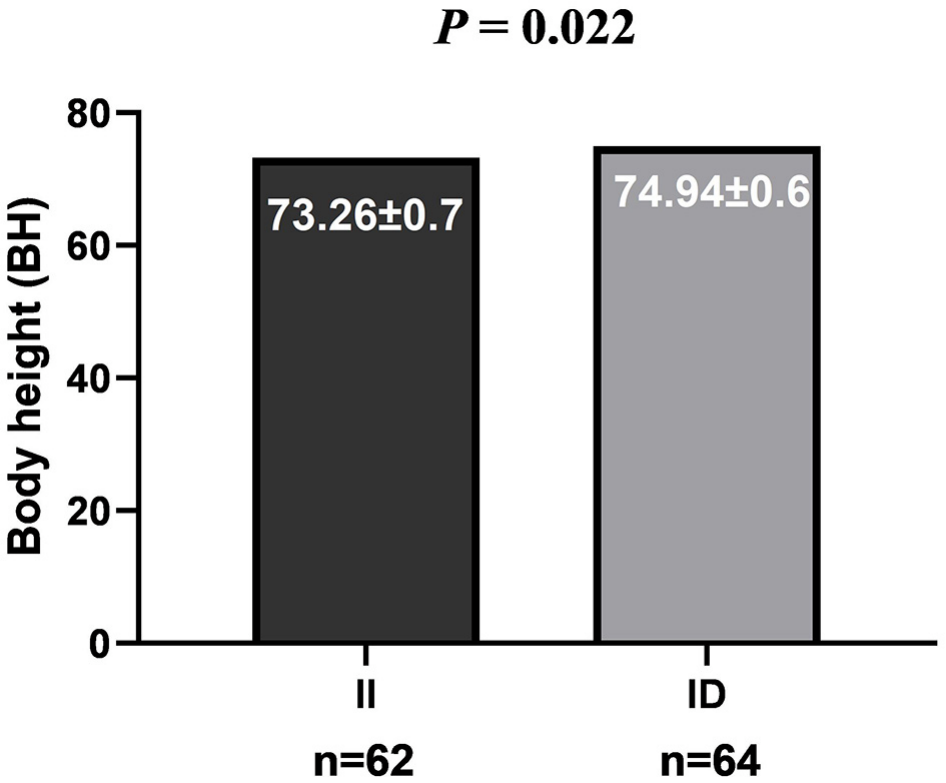
Association of the Del-90-bp with growth traits in weaner rams of the GQSFW sheep.

The genotype analysis of 90-bp deletion showed associations with growth traits in LXBH and GQSFW flocks. When correcting for the test of phenotypes using the Bonferroni correction, there was no significant difference between 90-bp indel and growth traits in the LXBH sheep groups (Table 3). However, associations were found between 90-bp deletion polymorphism and several growth traits (body length, body height, chest depth, and canon circumference) in GQSFW ewe adults after the Bonferroni correction (*P* < 0.0002), and individuals with the “DD” genotypes had greater growth traits (Figure 1). Meanwhile, after the Bonferroni correction, a significant association did not exist in GQSFW ram weaners (Figure 2). Besides, we did not detect associations between these two variations and growth traits in LZFT and WN samples, which might be due to the small sample size.

**Table 3:** Association of the Del-90-bp with growth traits in LXBH sheep.

**Gender**	**Age**	**Growth traits**	**Observed genotypes**		***P-*values**
			**II**	**ID**	
Ram	Lamb	BW (kg)	24.20 ± 2 (*n* = 15)	33.11 ± 1 (*n* = 19)	0.010
		ChD (cm)	30.63 ± 1 (*n* = 18)	27.95 ± 0.6 (*n* = 23)	0.039
		ChCI (cm)	74.80 ± 1 (*n* = 18)	74.44 ± 1 (*n* = 23)	0.044
Ewe	Lamb	ChW (cm)	14.78 ± 0.4 (*n* = 43)	16.0 ± 0.3 (*n* = 38)	0.026
		CaC (cm)	7.6 ± 0.1 (*n* = 43)	8.1 ± 0.1 (*n* = 38)	0.015
		HW (cm)	13.56 ± 0.3 (*n* = 43)	15.07 ± 0.3 (*n* = 38)	0.008
		ChCI	25.65 ± 0.6 (*n* = 43)	27.63 ± 0.4 (*n* = 38)	0.020
		CaCI	13.31 ± 0.2 (*n* = 43)	14.06 ± 0.2 (*n* = 38)	0.030
	Adult	BW (kg)	55.81 ± 2 (*n* = 16)	46 ± 3 (*n* = 7)	0.022
		HW (cm)	9.5 ± 1 (*n* = 27)	9.3 ± 1 (*n* = 37)	0.016

BW, body weight; BTI, body trunk index; ChD, chest depth; ChW, chest width; ChCI, chest circumference index; CaCI, cannon circumference index; CaC, cannon circumference; LLI, limb length index; HW, hip width. With the *p*-value corrected for multiple comparisons (α_altered_ = *P*/n, where *P* = 0.05, n = number of sheep) and after the Bonferroni correction, no association withstands (*P* < 0.001; *P* < 0.0006; *P* < 0.0007; *P* < 0.002). Genotypes were omitted because the number size was less than three or not detected.

## 4. Discussion

The knowledge of growth patterns and their use in managing the development of animals is an additional reserve for improving their productivity and livestock production (24). The results of the correlation analysis and the determination of the relationships between body performance indicators enable researchers to make optimal decisions on the selection of growth traits in the specialized sheep breeds (25, 26).

To our knowledge, this is the first study that demonstrates the influence of p.Q249R and 90-bp deletion polymorphisms on growth traits in the LXBH and GQSFW sheep. Following the outcomes of the association analysis, individuals carrying the wild-type (++) genotype showed better growth traits in LXBH ewe lambs than those with heterozygous (B+) genotypes. Our study outcomes were in agreement with results found in GWAS, which revealed that SNP in the *BMPRIB* gene was significantly related to sheep body weight and body slanting length (17). The study conducted by Sejian et al. (27) showed that the ewe lambs who were non-carriers of Garole × Malpura (GM) had significantly higher body measurement traits compared to those with homozygous mutant and heterozygous genotypes. Meanwhile, Kumar et al. (12) reported that the body weights of non-carriers (++) were higher than those of the carriers (BB and B+) in GM crossbreed. The Garole × Malpura breed has been obtained by crossing a highly prolific Garole breed with the Malpura mutton sheep breed (12). In addition, Gootwine et al. (18) also found that the FecB mutation affects the birth and mature weights of ewes. On the one hand, the body weight and morphometric traits of normally developed and well-fed animals might be influenced by the individual genetic characteristics of the breed. On the other hand, inadequate sample size may lead to biased results.

An association of 90-bp deletion with different genotypes was also found to be related to growth traits in LXBH and GQSFW sheep. In detail, individuals with DD or ID genotypes had greater growth traits compared to the individuals with II genotypes. It has been reported that ewes with the DD genotype had better litter size in Hu and East Friesian/Hu crossbred sheep in the study conducted by Li et al. (28). Notably, the same study found a 90-bp deletion in strong linkage to the causative g.746A>G SNP (28). Given that this locus is located in intron 1 of the *BMPRIB* gene, it is conceivable that it may influence gene expression through splicing or other specific events. Additionally, intronic deletions tend to be more common in the genome and can manifest significant changes in the gene locus length (29). Further comprehensive experiments are needed to prove the additional functions of this variant.

This is the first study that revealed an association between this variant and sheep body measurement traits. However, there were no strong associations after the Bonferroni correction in some sheep groups; however, the Bonferroni correction is generally stringent for genetic association analysis. Therefore, the detected associations might be real, as this research study was carried out using a large sample size. Further, there were no associations between these two variations and growth traits in LZFT and WN samples, which might be due to the small sample size.

A gene that controls the formation of a protein or enzyme may also influence the formation of useful traits due to its pleiotropic action. The pleiotropic action of a gene can also appear as a result of the secondary influence of a protein produced under its control on individual biochemical and physiological processes in the animal organism (30). According to modern scientific concepts, BMPs are multifunctional growth factors that play multiple roles in skeletal development, homeostasis, and regeneration. The primary function of BMPs is to support the process of bone formation in the adult body (31). Furthermore, the binding of BMP to BMPR leads to the phosphorylation of downstream Smad proteins, thereby triggering the intracellular signaling cascade (32). Interestingly, in recent decades, genetic studies in humans and mice have shown that BMP signaling disorders through BMPRI lead to various bone, cartilage, and muscle diseases (33, 34). For example, a patient with a homozygous mutation in *BMPRIB* has severe limb deformities consisting of short stature and additional genital anomalies (35). However, Yi et al. (36) argued that the *BMPRIB* gene had widespread overlapping functions with other BMP receptors because the *BMPRIB* and *BMP7* double mutants showed more severe skeletal defects than single *BMPRIB* knockout. Consequently, the mechanism of the *BMPRIB* gene polymorphisms affecting the sheep growth traits is worthy of further in-depth exploration.

## 5. Conclusion

Briefly, after correcting for multiple comparisons (Bonferroni correction), the p.Q249R and 90-bp genotypes remained strongly associated with growth traits in LXBH and GQSFW sheep. Nevertheless, our results align with the experimental observations from GWAS, which identified the *BMPRIB* gene as a potential candidate gene for body measurement traits. These findings not only confirm the previous study's results but also expand on them. Therefore, further investigations regarding the effect of *BMPRIB* polymorphisms on growth traits are necessary for large sample sizes in other breeds.

## Data availability statement

The original contributions presented in the study are included in the article/Supplementary material, further inquiries can be directed to the corresponding author/s.

## Ethics statement

The animal studies were approved by Faculty Animal Policy and Welfare Committee of Northwest A&F University under contract (protocol no. NWAFU-314020038). The studies were conducted in accordance with the local legislation and institutional requirements. Written informed consent was obtained from the owners for the participation of their animals in this study.

## Author contributions

XL: Conceptualization, Funding acquisition, Supervision, Methodology, Project administration, Writing—review & editing. NA: Formal Analysis, Investigation, Validation, Writing—review & editing. HX: Conceptualization, Resources, Writing—review & editing. YL: Formal Analysis, Investigation, Methodology, Writing—review & editing. ZG: Resources, Writing—review & editing. CP: Conceptualization, Funding acquisition, Writing—review & editing. ES: Resources, Writing—review & editing. NM: Writing—review & editing, Formal Analysis. ZA: Conceptualization, Formal Analysis, Investigation, Validation, Writing—original draft, Writing—review & editing.

## References

[1] Akhatayeva Z Li H Mao C Cheng H Zhang G. Detecting novel Indel variants within the GHR gene and their associations with growth traits in Luxi Blackhead sheep. Anim Biotechnol. (2022) 33:214–22. 10.1080/10495398.2020.1784184 32615865

[2] Akhatayeva Z Mao C Jiang F Pan C Lin C Hao K. Indel variants within the PRL and GHR genes associated with sheep litter size. Reprod Domest Anim. (2020) 55:1470–8. 10.1111/rda.13796 32762057

[3] Mao C Akhatayeva Z Cheng H Zhang G Jiang F Meng X. A novel 23 bp indel mutation in PRL gene is associated with growth traits in Luxi Blackhead sheep. Anim Biotechnol. (2021) 32:740–7. 10.1080/10495398.2020.1753757 32293991

[4] Mo F Sun W Zhang L Zhang X La Y Xiao F. Polymorphisms in BMPRIB gene affect litter size in Chinese indigenous sheep breed. Anim Biotechnol. (2023) 34:538–45. 10.1080/10495398.2021.1980400 34570690

[5] Akhatayeva Z Bi Y He Y Khan R Li J Li H. Survey of the relationship between polymorphisms within the *BMPR1B* gene and sheep reproductive traits. Anim Biotechnol. (2023) 34:718–27. 10.1080/10495398.2021.1979023 34586970

[6] Zhou S Yu H Zhao X Cai B Ding Q Huang Y. Generation of gene-edited sheep with a defined Booroola fecundity gene (FecB^B^) mutation in bone morphogenetic protein receptor type 1B (BMPR1B) via clustered regularly interspaced short palindromic repeat (CRISPR)/CRISPR-associated (Cas) 9. Reprod Fertil Dev. (2018) 30:1616–21. 10.1071/RD18086 31039970

[7] Wen YL Guo XF Ma L Zhang XS Zhang JL Zhao SG. The expression and mutation of BMPR1B and its association with litter size in small-tail Han sheep (*Ovis aries*). Arch Anim Breed. (2021) 64:211–21. 10.5194/aab-64-211-2021 34109270 PMC8182661

[8] Al-Barzinji YMS. Polymorphism in Booroola (FecB) gene associated with litter size in Hamdani sheep. The 3^rd^ Kurdistan conference on biological science. J Dohuk Univ. (2015) 13:413–7.

[9] Kumar S Kolte AP Mishra AK Arora AL Singh VK. Identification of the FecB mutation in Garole × Malpura sheep and its effect on litter size. Small Rumin Res. **(**2006) 64:305–10. 10.1016/j.smallrumres.2005.04.030

[10] Wang W La Y Zhou X Zhang X Li F Liu B. The genetic polymorphisms of TGFβ superfamily genes are associated with litter size in a Chinese indigenous sheep breed (Hu sheep). Anim Reprod Sci. (2018) 189:19–29. 10.1016/j.anireprosci.2017.12.003 29274749

[11] Polley S De S Brahma B Mukherjee A Vinesh PV Batabyal S. Polymorphism of BMPR1B, BMP15 and GDF9 fecundity genes in prolific Garole sheep. Trop Anim Health Prod. (2010) 42:985–93. 10.1007/s11250-009-9518-1 20020203

[12] Kumar S Mishra AK Kolte AP Arora AL Singh D Singh VK. Effects of the Booroola (FecB) genotypes on growth performance, ewe's productivity efficiency and litter size in Garole × Malpura sheep. Anim Reprod Sci. (2008) 105:319–31. 10.1016/j.anireprosci.2007.03.012 17449205

[13] Wilson T Wu X-Y Juengel JL Ross IK Lumsden JM Lord EA. Highly prolific Booroola sheep have a mutation in the intracellular kinase domain of bone morphogenetic protein IB receptor (ALK-6) that is expressed in both oocytes and granulosa cells. Biol Reprod. (2001) 64:1225–35. 10.1095/biolreprod64.4.1225 11259271

[14] Wang X Guo X He X Liu Q Di R Hu W. Effects of *FecB* mutation on estrus, ovulation, and endocrine characteristics in Small Tail Han sheep. Front Vet Sci. (2021) 8:709737. 10.3389/fvets.2021.709737 34881317 PMC8646036

[15] Chu M Jia L Zhang Y Jin M Chen H Fang L. Polymorphisms of coding region of BMPR-IB gene and their relationship with litter size in sheep. Mol Biol Rep. (2011) 38:4071–6. 10.1007/s11033-010-0526-z 21110108

[16] Zhang CS Geng LY Du LX Liu ZZ Fu ZX Feng MS. Polymorphic study of FecXG, FecGH and FecB mutations in four domestic sheep breeds in the lower yellow river valley of China. J Anim Vet Adv. (2011) 10:2198–201. 10.3923/javaa.2011.2198.2201

[17] Li X He SG Li WR Luo L-Y Yan Z Mo D-X. Genomic analyses of Pamir argali, Tibetan sheep, and their hybrids provide insights into chromosome evolution, phenotypic variation, and germplasm innovation. Genome Res. (2022) 32:1669–84. 10.1101/gr.276769.122 35948368 PMC9528982

[18] Gootwine E Rozov A Bor A Reicher S. Carrying the FecB (Booroola) mutation is associated with lower birth weight and slower post-weaning growth rate for lambs, as well as a lighter mature bodyweight for ewes. Reprod Fertil Dev. (2006) 18:433–7. 10.1071/RD05134 16737636

[19] Oraon T Singh DK Ghosh M Kullu SS Kumar R Singh LB. Allelic and genotypic frequencies in polymorphic Booroola fecundity gene and their association with multiple birth and postnatal growth in Chhotanagpuri sheep. Vet World. (2016) 9:1294–9. 10.14202/vetworld.2016.1294-1299 27956784 PMC5146313

[20] Guan F Liu SR Shi GQ Yang LG. Polymorphism of FecB gene in nine sheep breeds or strains and its effects on litter size, lamb growth and development. Anim Reprod Sci. (2007) 99:44–52. 10.1016/j.anireprosci.2006.04.048 16859845

[21] Akhatayeva Z Cao C Huang Y Zhou Q Zhang Q Guo Z. Newly reported 90-bp deletion within the ovine BMPRIB gene: does it widely distribute, link to the famous FecB (p.Q249R). Theriogenology. (2022) 189:222–9. 10.1016/j.theriogenology.2022.06.020 35785581

[22] Bolormaa S Pryce JE Reverter A Zhang Y Barendse W Kemper K. A multi-trait, meta-analysis for detecting pleiotropic polymorphisms for stature, fatness and reproduction in beef cattle. PLoS Genet. (2014) 10:e1004198. 10.1371/journal.pgen.1004198 24675618 PMC3967938

[23] Davis GH Balakrishnan L Ross IK Wilson T Galloway SM Lumsden BM. Investigation of the Booroola (FecB) and Inverdale (FecXI) mutations in 21 prolific breeds and strains of sheep sampled in 13 countries. Anim Reprod Sci. **(**2006) 92:87–96. 10.1016/j.anireprosci.2005.06.001 15982834

[24] Pan Y Wang M Wu H Akhatayeva Z Lan X Fei P. Indel mutations of sheep PLAG1 gene and their associations with growth traits. Published online ahead of print. Anim Biotechnol. (2021) 33:1459–65. 10.1080/10495398.2021.1906265 33825658

[25] Li X Yang J Shen M Xie X-L Liu G-J Xu Y-X. Whole-genome resequencing of wild and domestic sheep identifies genes associated with morphological and agronomic traits. Nat Commun. (2020) 11:2815. 10.1038/s41467-020-16485-1 32499537 PMC7272655

[26] Chong Y Liu G Jiang X. Effect of BMPRIB gene on litter size of sheep in China: a meta-analysis. Anim Reprod Sci. (2019) 210:106175. 10.1016/j.anireprosci.2019.106175 31635771

[27] Sejian V Maurya VP Prince LLL Kumar D Naqvi SMK. Effect of FecB status on the allometric measurements and reproductive performance of Garole × Malpura ewes under hot semi-arid environment. Tropical Anim Health Prod. (2015) 7:1089–93. 10.1007/s11250-015-0831-6 25911004

[28] Li D Zhang L Wang Y Chen X Li F Yang L. *FecB* mutation and litter size are associated with a 90-base pair deletion in *BMPR1B* in East Friesian and Hu crossbred sheep. Anim Biotechnol. (2022) 34:1314–23. 10.1080/10495398.2021.2020805 34985398

[29] Vaz-Drago R Custódio N Carmo-Fonseca M. Deep intronic mutations and human disease. Hum Genet. (2017) 136:1093–111. 10.1007/s00439-017-1809-4 28497172

[30] Watanabe K Stringe S Frei O Umićević Mirkov M de Leeuw C Polderman TJC. A global overview of pleiotropy and genetic architecture in complex traits. Nat Genet. (2015) 51:1339–48. 10.1038/s41588-019-0481-0 31427789

[31] Stantzou A Schirwis E Swist S Alonso-Martin S Polydorou I Zarrouki F. BMP signaling regulates satellite cell-dependent postnatal muscle growth. Development. (2017) 144:2737–47. 10.1242/dev.144089 28694257 PMC5560039

[32] Miyazawa K Miyazono K. Regulation of TGF-β family signaling by inhibitory smads. Cold Spring Harb Perspect Biol. (2017) 9:a022095. 10.1101/cshperspect.a022095 27920040 PMC5334261

[33] Wang RN Green J Wang Z Deng Y Qiao M Peabody M. Bone Morphogenetic Protein (BMP) signaling in development and human diseases. Genes Dis. (2014) 1:87–105. 10.1016/j.gendis.2014.07.005 25401122 PMC4232216

[34] Wu M Chen G Li YP. TGF-β and BMP signaling in osteoblast, skeletal development, and bone formation, homeostasis and disease. Bone Res. **(**2006) 4:16009. 10.1038/boneres.2016.9 27563484 PMC4985055

[35] Demirhan O Türkmen S Schwabe GC Soyupak S Akgül E Tastemir D. A homozygous BMPR1B mutation causes a new subtype of acromesomelic chondrodysplasia with genital anomalies. J Med Genet. (2005) 42:314–7. 10.1136/jmg.2004.023564 15805157 PMC1736042

[36] Yi SE LaPolt PS Yoon BS Chen JYC Lu JKH Lyons KM. The type I BMP receptor BmprIB is essential for female reproductive function. Proc Natl Acad Sci USA. (2001) 98:7994–9. 10.1073/pnas.141002798 11416163 PMC35456

